# Stem Cell Therapy: The Eventual Future

**DOI:** 10.4103/0974-7753.58562

**Published:** 2009

**Authors:** Rajiv Saini, Santosh Saini, Sugandha Sharma

**Affiliations:** Department of Periodontology and Oral Implantology, Rural Dental College - Loni, India; 1 Department of Microbiology, Rural Dental College - Loni, India; 2 Department of Prosthodontics, Rural Dental College - Loni, India

Sir,

Stem cells are unspecialized cells that develop into the specialized cells that make up different types of tissue in the human body. They are vital to the development, growth, maintenance, and repair of our brains, bones, muscles, nerves, blood, skin, hair, and other organs. The isolation, culture, and partial characterization of stem cells isolated from human embryos were reported in the November of 1998.[1] The hair follicle bulge possesses putative epithelial stem cells and genetic profiling of hair follicle stem cells revealed several known and unknown receptors and signaling pathways important for maintaining the stem cell phenotype. Ultimately, these findings provide potential targets for the treatment of hair loss and other disorders of the skin and hair.[2] Stem cell therapy is a treatment that uses stem cells, or cells that come from stem cells, to replace or to repair a patient's cells or tissues that are damaged. The stem cells might be put into the blood or transplanted into the damaged tissue directly or even recruited from the patient's own tissues for self-repair. Stem cells are vital for the homeostasis of self-renewing tissues such as the hair follicle.[3]

Stem cell technologies have, or are anticipated to have, applications for basic science (study of complex processes), medical research (to produce large numbers of genetically uniform cultures of organ tissues-for example, liver, muscle, or neural), and therapies (repair or replace damaged or diseased tissues). Experiments with mice and rats as well as preliminary work with humans have raised hopes about the eventual development of therapies using stem cells. Human stem cell research holds enormous potential for contributing to our understanding of fundamental human biology. The physiology as well as molecular and cellular biology of the ocular surface and its components has not only increased our understanding but has also opened a new chapter in regenerative medicine. With the use of tissue engineering, various scientific discoveries in the past two decades have led to the identification of the limbal location of corneal epithelial stem cells and the role it plays in regenerating the corneal epithelium.[4] Novel techniques of *ex vivo* epithelium cell culture have allowed us to treat limbal stem cell deficiency with a better surgical approach, which requires a very small donor tissue, obviating the need for immunosuppression, minimizing the risk to the donor site and increasing the possibility of obtaining an autologous donor tissue from a small, uninvolved area of the limbus, called autologous cultivated limbal epithelium transplantation.[4] Novel tissue-engineered corneas that comprise composites of natural and synthetic biopolymers together with corneal cell lines or stem cells will, in the future, replace portions of the cornea that are damaged and provide a basis for the development of both implantable temporary and permanent corneal replacements.[5] *In vitro* models using primary cultures of corneal epithelium and lines of corneal epithelial cells with an extended life span retain a variety of phenotypic characteristics and may be used as an adjunct to ocular toxicology studies and as a tool to investigate corneal epithelial cell biology.[6] In addition to the need for more data to further define the relationship between the epidermis and the hair follicle, many important issues need to be addressed.

More cell surface markers are clearly needed for the further enrichment and molecular characterization of keratinocyte stem cells (KSCs). It is known that, during wound repair, KSCs can be stimulated to divide and transit amplifying cells can increase the number of rounds of DNA replication that they exhibit.[7] The human hair follicle bulge is an important niche for KSCs and elucidation of human bulge cell biology could be facilitated by analyzing global gene expression profiles and identifying unique cell-surface markers. The lack of distinctive bulge morphology in human hair follicles has hampered studies of bulge cells and KSCs.[8] Innovative research and new technologies derived from such research almost always raise ethical and policy concerns. In biomedical research, these issues include the ethical conduct of basic and clinical research as well as the equitable distribution of new therapies. These issues are relevant to discussions about stem cell research and its eventual applications; however, they are part of a constellation of ethical and policy concerns associated with all advances in biomedical research. It is essential that there be a public that is educated and informed about the ethical and policy issues raised by stem cell research and its applications. Stem cell research and its applications hold scientific and medical promise. Like other powerful technologies, they pose challenges as well as risks. If we are to realize the benefits, meet the challenges, and avoid the risks, stem cell research must be conducted under effective, accountable systems of social oversight and control, at both the national and international levels. Stem cells offer opportunities for scientific advances that go far beyond regenerative medicine.
